# Rapid detection of SARS-CoV-2: The gradual boom of lateral flow immunoassay

**DOI:** 10.3389/fbioe.2022.1090281

**Published:** 2023-01-10

**Authors:** Jing He, Shuying Zhu, Jiawei Zhou, Wenjie Jiang, Liliang Yin, Lan Su, Xinling Zhang, Qi Chen, Xiaoping Li

**Affiliations:** Shulan International Medical College, Zhejiang Shuren University, Hangzhou, China

**Keywords:** SARS-CoV-2, lateral flow immunoassay, nanotechnology, CRISPR, COVID-19, antibody, antigen, nucleic acid

## Abstract

Severe Acute Respiratory Syndrome Coronavirus 2 (SARS-CoV-2) is still in an epidemic situation, which poses a serious threat to the safety of people and property. Rapid diagnosis and isolation of infected individuals are one of the important methods to control virus transmission. Existing lateral flow immunoassay techniques have the advantages of rapid, sensitive, and easy operation, and some new options have emerged with the continuous development of nanotechnology. Such as lateral flow immunoassay test strips based on colorimetric-fluorescent dual-mode and gold nanoparticles, Surface Enhanced Raman Scattering, etc., these technologies have played an important role in the rapid diagnosis of COVID-19. In this paper, we summarize the current research progress of lateral flow immunoassay in the field of Severe Acute Respiratory Syndrome Coronavirus 2 infection diagnosis, analyze the performance of Severe Acute Respiratory Syndrome Coronavirus 2 lateral flow immunoassay products, review the advantages and limitations of different detection methods and markers, and then explore the competitive CRISPR-based nucleic acid chromatography detection method. This method combines the advantages of gene editing and lateral flow immunoassay and can achieve rapid and highly sensitive lateral flow immunoassay detection of target nucleic acids, which is expected to be the most representative method for community and clinical point-of-care testing. We hope that researchers will be inspired by this review and strive to solve the problems in the design of highly sensitive targets, the selection of detection methods, and the enhancement of CRISPR technology, to truly achieve rapid, sensitive, convenient, and specific detection of novel coronaviruses, thus promoting the development of novel coronavirus diagnosis and contributing our modest contribution to the world’s fight against epidemics.

## 1 Introduction

2019 Coronavirus disease (COVID-19) is an infectious disease caused by Severe Acute Respiratory Syndrome Coronavirus 2 (SARS-CoV-2) (2022a). The once-in-a-century pandemic epidemic caused by COVID-19 (2020c) has taken a huge toll on lives and livelihoods, disrupting health systems, economies, and societies (2020b). According to WHO, as of 2 September 2022, there have been more than 600 million confirmed cases of COVID-19 worldwide, including more than 6.47 million deaths (2022e).

**FIGURE 1:**
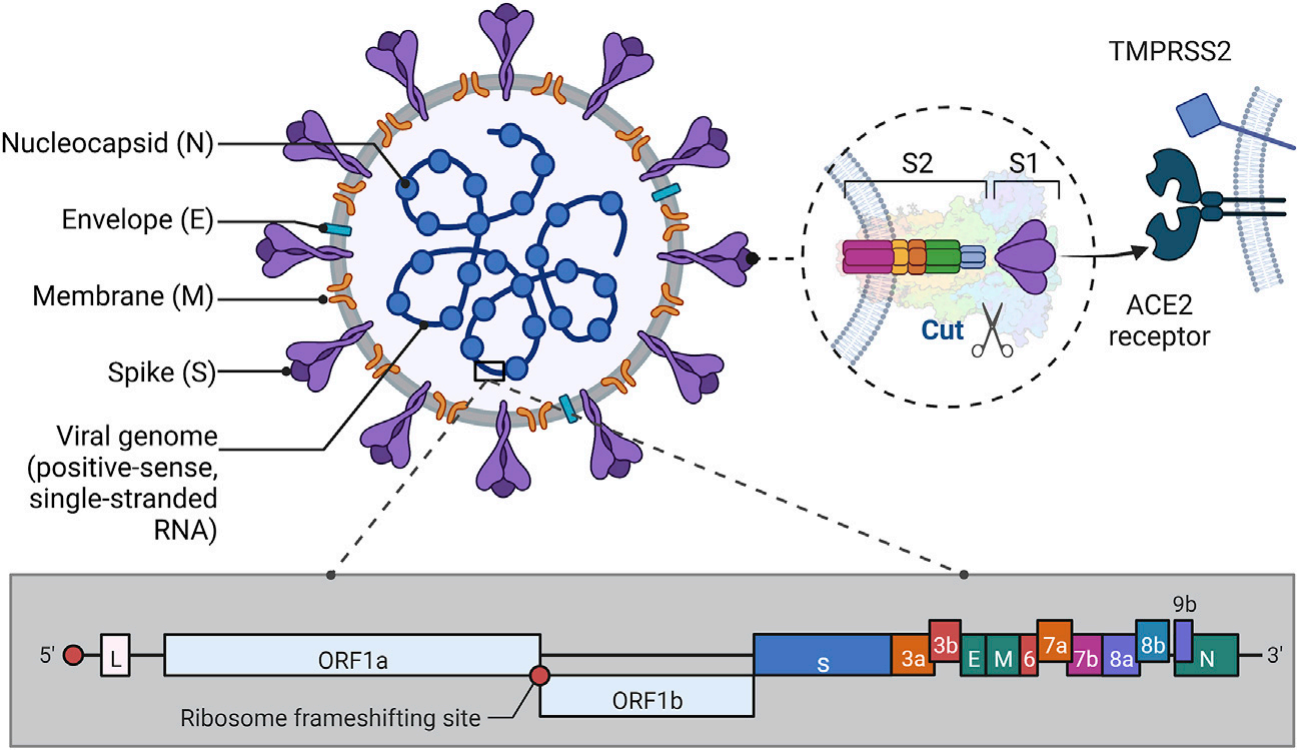
Structure of SARS-CoV-2. Illustrative scheme: This figure shows the approximate protein structure of SARS-CoV-2 and the gene fragment of RNA, in addition to depicting the way S1 binds to the ACE2 receptor to invade the organism. As can be seen in the figure, except for the N protein which is wrapped around the nucleic acid, the E, M, and S proteins are anchored to the envelope, each in its way. Among them, the S protein consists of two subunits, S1 and S2. S1 protein has a receptor binding domain (RBD) on it that binds to ACE2 on the host cell. S2 protein has a more complex structure and its role is to fuse the virus to the host cell membrane. In the pre-fusion conformation, the S1 and S2 subunits remain non-covalently bound. When the novel coronavirus wants to enter the host cell, the S protein shifts from the closed to the open state, thus mediating entry into the host cell (Lan et al., 2020; Shang et al., 2020; Walls et al., 2020). In addition, when RBD is structured and ACE2, it is activated by proteases such as TMPRSS2 on the host cell membrane, which causes the S1/S2 enzymatic cleavage site to be cut, thus facilitating the fusion of the virus with the host cell membrane (Zhou et al., 2020). The genetic structure of the virus mentioned in the paper is also briefly shown in Fig. The RNA genome of SARS-CoV-2 consists of 14 open reading frames (ORFs) (Khailany et al., 2020). Where ORF1a and ORF1b overlap at the (-1) ribosomal frameshift, this part contains about two-thirds of the genome and is mainly used for processing and synthesis of non-borrowed proteins. The remaining one-third of the genome in which the different ORFs also overlap each other is mainly used to encode four structural proteins (Arya et al., 2021). (This figure created with BioRender.com).

SARS-CoV-2 is a betacoronavirus, an enveloped virus containing a large nucleoprotein-encapsidated positive sense RNA genome (Ke et al., 2020). SARS-CoV-2 contains four structural proteins: spike (S), membrane (M), nucleocapsid (N), and envelope (E) proteins. The N protein is mainly involved in RNA packaging and virus particle release and is a highly immunogenic and abundantly expressed protein during the infection process and is therefore commonly used in serotype analysis (Zeng et al., 2020; Liu et al., 2006); the S protein exhibits virus specificity mediates virus-host cell adhesion and invades the organism by recognizing and binding angiotensin-converting enzyme 2 (ACE2) (Wang et al., 2020; V'Kovski et al., 2021; Kadam et al., 2021). The process is illustrated in the following figure (Figure 1). The organization of the SARS-CoV-2 genomeis 5′-leader-UTR- replicase-S (Spike)-E (Envelope)-M (Membrane)-N (Nucleocapsid)-3′UTR-poly (A) tail with accessory genes interspersed within the structural genes at the 3′end of the genome (Malik, 2020). After infection with SARS-CoV-2, an immune response occurs in the human body to produce immunoglobulins corresponding to the antigen. Studies have shown that SARS-CoV-2 is highly infectious (Harrison et al., 2020), and the production of variants (Harvey et al., 2021; Tao et al., 2021) and the increase in asymptomatic infections (Johansson et al., 2021; Tanriover et al., 2021) have growth its transmission rate. Infection with SARS-CoV-2 affects multiple organs, as ACE2 is present in large numbers in human lung and small intestine epithelial cells, making the respiratory (Mandal et al., 2021; Stavem et al., 2021) and digestive (Aiyegbusi et al., 2021) systems susceptible to viral attack and disease, and it has also been documented that ACE2 is also expressed in the bile ducts, thus predisposing to liver injury (Hamming et al., 2004; Yang et al., 2020). Besides, ACE 2/angiotensin-(1–7)/MAS can counteract the negative effects of the renin-angiotensin system, however, ACE 2 downregulation after infection can lead to multi-organ damage, such as cardiovascular (Dweck et al., 2020; Puntmann et al., 2020) and neurological (Moghimi et al., 2021) diseases, among others (Ni et al., 2020). Currently, the gold standard for the detection of SARS-CoV-2 is real-time reverse transcription-polymerase chain reaction (RT-PCR), which has high specificity and relatively high sensitivity and accuracy of detection, and can accurately diagnose the disease during the latent phase and determine the virulence and mutation site of the virus, and has an irreplaceable position in the diagnosis of COVID-19 (Corman et al., 2020; Hoehl et al., 2020; Rothe et al., 2020; Kampf et al., 2021; Rahbari et al., 2021). However, it is time-consuming and requires high equipment and personnel, which makes the virus spread much faster than the diagnosis and isolation, and cannot meet the requirements of rapid detection in epidemic prevention and control (Safiabadi Tali et al., 2021b; Gupta et al., 2021). Therefore, there is an urgent need to develop a rapid detection method for COVID-19 to effectively curb the spread of the epidemic.

Lateral flow immunoassay (LFIA) is an assay developed by applying the principles of chromatographic chromatography and specific binding of antigen and antibody (Nguyen et al., 2020). LFIA strips consist of a sample pad, a releasing pad, nitrocellulose (NC) membrane, an absorbent pad, and a polyvinyl chloride backing. The principle is that after the sample is added to the sample pad, the test material binds to the nano marker on the releasing pad by capillary action, thus forming a coupling and binding to the corresponding ligand in the detection line on the NC membrane to show changes in color or light, which is used for qualitative or quantitative detection of SARS-CoV-2, and finally, the remaining liquid reaches the aspiration pad (Islam and Iqbal, 2020; Mahmoudinobar et al., 2021). Some studies have shown that LFIA still has advantages over the gold standard, such as rapid and accurate detection of patients in the period of shedding virus, and can be used for primary screening with the advantage of its rapid detection, thus reducing the waste of resources, under the full consideration of diagnostic accuracy, which also depends on various factors such as time to result, acceptable complexity of the test, and acceptance by the tested person (Dinnes et al., 2020; Yamayoshi et al., 2020). However, the performance of LFIA is definitely inferior to that of the gold standard, and researchers need to make efforts to shorten the detection window and improve the specificity to promote the development of LFIA testing (Tuaillon et al., 2020). Since Faulk (Faulk and Taylor, 1971) pioneered LFIA technology, it has been widely used in many fields such as food testing (Xu et al., 2021) and microbial detection (Zeng et al., 2021). With the continuous development of nanotechnology, the LFIA technology has also been extended with different detection modes, which can be classified into colloidal gold nanoparticle-based immunoassay (Qin et al., 2021), automated fluorescence immunoassay (Kang et al., 2021b), magnetic particle immunoassay (Bai et al., 2020), etc. according to the different markers.

After the outbreak of COVID-19, LFIA technique has taken the advantage in the rapid detection of COVID-19 because of its rapid, cheap, and convenient operation (Porte et al., 2020), which can effectively help control the epidemic and cut off the infection chain; it can also solve the problem that many countries cannot perform large-scale PCR detection due to the backward technology (2020a). At present, LFIA has been widely used in the detection of antigens (Pollock et al., 2021), antibodies (Fulford et al., 2021), and nucleic acid (Zou et al., 2021) of SARS-CoV-2 according to different detection targets, and some LFIA test strips have been marketed and used. In addition, new nanotechnologies are being applied to LFIA to improve the specificity and sensitivity of COVID-19 detection. In this paper, we will review the applications of LFIA combined with nanotechnology for the rapid detection of SARS-CoV-2 antigen, antibody, and nucleic acid based on LFIA and summarize the different detection modes; we will also outline and compare the LFIA products for the rapid detection of SARS-CoV-2 in China and abroad; Additionally, we will summarize the development of LFIA for the rapid detection of SARS-CoV-2. Finally, the development of LFIA for rapid SARS-CoV-2 detection is summarized.

## 2 LFIA technique for SARS-CoV-2 antibody detection

Antibodies are immunoglobulins produced by B cells or plasma cells by the immune system under the stimulation of antigens that bind specifically to antigens and are mainly classified into five categories: immunoglobulin M (IgM), immunoglobulin G (IgG), immunoglobulin A (IgA), immunoglobulin D (IgD) and immunoglobulin E (IgE) (Post et al., 2020). Therefore, after the invasion of a pathogen microorganism, the antibodies produced can be used as biomarkers for LFIA technology to determine whether it is infected. For example, its use in the diagnosis of pathogenic microbial infections such as Coccidioides (Azeem et al., 2022), *Chlamydia trachomatis* (Gwyn et al., 2019), and West Nile virus (Rebollo et al., 2018) has had many applications, so LFIA techniques based on SARS-CoV-2 antibodies for the diagnosis of COVID-19 have been widely followed.

### 2.1 Colloidal gold immunoassay technique for SARS-CoV-2 antibody detection

Colloidal gold immunoassay is the traditional LFIA technique, which uses gold nanoparticles as markers and is most widely used in SARS-CoV-2 antibody detection. Colorimetric analysis is a method that uses the color of the measured solution itself, or the color presented by the addition of reagents, to determine the concentration of the measured substance in the solution by observing and comparing the color depth of the solution by eye (or visual colorimeter), or by measuring it with a photoelectric colorimeter. The colorimetric method includes visual colorimetry and photoelectric colorimetry (Fernandes et al., 2020). Visual colorimetry is the most direct and simple method to determine the negative or positive diagnostic result in LFIA. For example, the following two individuals performed the test using the visual colorimetric method. Wang et al. (Wang et al., 2021a) designed a colloidal gold immunoassay kit for the detection of two antibodies, IgM and IgG, to the SARS-CoV-2 N protein. The colloidal gold of their test strips was modified with SARS-CoV-2 N antigen obtained from the expression system of pCMVp-NEO-BAN vector + HEK293 cells, and mouse anti-human IgM and IgG were immobilized on the NC membrane. When the results were positive, colloidal gold SARS-CoV-2 recombinant antigen-antibody complexes bound to IgM or IgG detection lines and showed a purplish red color. The study detected a total of 128 SARS-CoV-2 positive and negative cases, and the samples were taken from the serum of the subjects, and the final sensitivity and specificity were found to be 88.66% and 90.63%, respectively. In addition, 1061 non-SARS-CoV-2 infected persons aged 21–71 years were analyzed, and the false positive rates of IgM and IgG obtained were .75% and 0%, respectively. Cavalera et al. (2021) designed a double detection line of colloidal gold immunoassay test strips for N protein IgA, IgM, and IgG codetection. The tested antibody extracted from serum on the releasing pad binds to the nanogold particles labeled with N protein and biotin, then sequentially passes through the detection line with SPA and N protein fixed and binds to it, then binds to biotin and avidin on the quality control line, and finally completes the detection by the naked eye and portable scanner within 20 min. The test strips were analyzed from 147 clinical samples (62 RT-PCR positive samples and 85 RT-PCR negative samples), resulting in diagnostic sensitivity and specificity of 94.6% and 100%, respectively. Thus, it seems that the performance of the visual colorimetric method for the detection of SARS-CoV-2 antibodies is good, and from the preparation method, the preparation method of LFIA kits based on colloidal gold are more or less the same, and the main difference in the performance of the above two groups of products is the substances labeled on colloidal gold. The former N antigen is obtained by pCMVp-NEO-BAN vector expression, and the latter by pSER vector expression, both are commonly used expression vectors, which can stabilize and highly express the antigen of the target gene, while the difference between the two needs to be further explored by scholars (Rosati et al., 2004). The latter also uses biotin-labeled nanogold, which can induce aggregation of colloidal particles and thus improve the specificity of the assay, so this can also be considered as one of the reasons for the more advantageous performance of the LFIA kit designed by Cavalera et al. and worthy of study by researchers.

Chemiluminescence immunoassay (CLIA) is a kind of labeled antibody technology, which labels antibodies or antigens with substances such as chemiluminescent agents, catalytic luminescent enzymes, or products indirectly involved in the luminescence reaction, etc. When the labeled antibody or labeled antigen binds to the corresponding antigen or antibody, the luminescent substrate is affected by the luminescent agent, catalytic enzymes, or products involved, and a redox reaction occurs, in which visible light is released or the reaction excites fluorescent substances to emit light, and finally detected by spectrophotometer (Oishee et al., 2021). Roda et al. (2021) reported an optical/chemiluminescence-based colloidal gold immunoassay test strip for the SARS-CoV-2 IgA’s semi-quantitative detection. The test strip was immobilized with N protein antigen on the detection line and staphylococcal protein A (SPA) on the quality control line. The optical method uses gold nanoparticles modified with mouse anti-human IgA to label the antibody and a smartphone camera to image the color band formed at the detection line for signal transduction and quantification. The chemical method uses mouse anti-human IgA modified horseradish peroxidase to label the antibody and a portable device with a CCD camera to capture and analyze the chemiluminescent signal. The method can be performed on saliva or serum samples and results can be obtained within 15 min. Ten SARS-CoV-2 negative samples and 25 positive serum samples were compared with RT-PCR, and the false positive and false negative rates were 0% and 40%, respectively, besides the sensitivity of the chemiluminescence assay was 10 times higher than that of the optical method.

Surface-enhanced Raman spectroscopy scattering (SERS) originates from the presence of a strong local plasma electromagnetic field on a rough surface at the nanoscale, and when molecules are adsorbed on the surface of a metal structure their scattering cross section is dramatically amplified by the local electromagnetic field of the metal surface, resulting in a 10^10^ to 10^11^fold increase in the Raman intensity of the molecules (Camden et al., 2008). Compared with gold nanoparticle-based colorimetric methods, the SERS immunochromatographic technique takes advantage of the high sensitivity and accuracy of SERS, allowing for a sensitivity increase of typically 3 to 4 orders of magnitude (Wang et al., 2017; Deng et al., 2021). SERS method is often combined with Au or Ag (Sultangaziyev et al., 2022). For example, Chen et al. (2021c) developed a surface-enhanced Raman scattering based on the LFIA strip to simultaneous detect IgM and IgG. The test strips require serum as the sample, and use 4-nitrobenzenethiol modified Au-conjugated COVID-19 recombinant antigen as gap-enhanced Raman nanotags, and mouse anti-human IgM, mouse anti-human IgG, and sheep anti-chicken IgY as two detection lines and one quality control line, respectively. Compared with the conventional nanotags, the surface-enhanced Raman scattering signal of gap-enhanced Raman nanotags was improved by 30-fold, and the sensitivity of the measured IgM and IgG was excellent, 1 nm/ml and .1 nm/ml, respectively. Liu et al. (Liu H. et al., 2021a) proposed a LFIA test strip based on the SiO2@Ag SERS label for the quantitative detection of IgM/IgG antibodies to the S protein of SARS-CoV-2. The nano-marker was fabricated using the DTNB modification method, and the bilayer DTNB-modified SiO2@Ag nanoparticles were made by redox method after coating nanogold on silicon monoxide, and then attached to S protein to capture the antibody. The marker exhibited excellent SERS signal, high stability, and good monodispersity. The SERS signal intensity of the SiO2@Ag-spike protein-anti-novel coronavirus IgM/IgG immunocomplex formed by the capture on the detection line can be recorded by a portable Raman instrument. The method can be performed on serum samples and yield results within 25 min. The researchers obtained 100% accuracy and specificity by testing 49 SARS-CoV-2 negative samples against 19 positive serum samples and comparing them with RT-PCR, and the detection limit was 1 pg/ml, which is 800 times more sensitive than the conventional colloidal gold-based LFIA method. From the experimental results of the researchers, the LFIA strips have more excellent sensitivity and specificity mainly attributed to the study of bilayer DTNB-modified SiO2@Ag nanoparticles, and the SERS signal is greatly increased after the bilayer DTNB modification and the surface attachment of Ag coating, so this method can also be studied in the design of markers for the SERS method in the future. Further analysis, scholars can also be inspired by the screening of different nanoparticle platforms for the SERS method. These different nanoparticle fabrication methods can be used to evolve the structure by changing the particle shape or designing different materials or platforms to achieve the ultimate goal of signal enhancement, and thus the method of Liu et al. is superior to the experimental results (Serafinelli et al., 2022). In addition, there are various ways to design probes by the SERS method, such as double DTNB modified magnetic SERS probes with the structure of Fe3O4/DTNB@Ag/DTNB, or SERS probes prepared by immobilizing the Raman reporter molecule of malachite green isothiocyanate or coupling 4- amino thiophenol to the surface of the Raman reporter molecule to form SERS probes, etc (Huang et al., 2020). All of these probes showed good sensitivity and can provide researchers with a reference in LFIA-based detection of new coronaviruses.

With the increasing number of people receiving the COVID-19 vaccine, there is an emerging trend for antibody testing for SARS-CoV-2 to assess vaccine efficacy. Liu et al. (2021b) also developed a colloidal gold immunoassay strip for combined lgM-lgG detection, which was used to test the effect of antibodies in vaccine recipients and thus evaluates the effectiveness of inactivated neocoronavirus vaccine. In this method, goat anti-human IgG and IgM were immobilized on the NC membrane, and goat anti-mouse IgG, S protein and N protein were modified on the surface of colloidal gold. The blood of the subject was taken, and if it produced IgG and IgM of S protein, and N protein due to vaccination, then when detected, the antibody combined with S protein, and N protein on the surface of colloidal gold, and then passed through the IgG and IgM detection line successively, and combined with the corresponding antibodies to form protein complexes, and finally the quality control line combined with the colloidal gold particles modified with goat anti-mouse IgG, so that both the detection line and the quality control line appeared red. The method was used to test clinical samples from three inactivated vaccine recipients and one non-inactivated vaccine recipient. The small sample size makes this study limited, but it provides a reference for future evaluation of vaccine efficacy.

### 2.2 SARS-CoV-2 antibody detection method based on other nanomaterials

With the continuous development of nanotechnology, many novel materials have been used for LFIA and have shown good sensitivity and specificity. In addition to traditional fluorescent nanoparticles, materials such as quantum dots and dye-loaded polymers are becoming increasingly popular (Banga Ndzouboukou et al., 2021).

Immunofluorescence techniques are based on the principle of antigen-antibody reaction, in which a known antigen (or antibody) labeled with a fluorescent moiety is first used as a probe to capture the corresponding antibody (or antigen) and thus localize the antibody (or antigen) to be measured using a fluorescent detector (Im et al., 2019). Traditional fluorescence-based LFIA techniques commonly use fluorescent latex particles as markers, as in the study conducted by Kang et al. (2021a) The investigators designed a kit for the co-testing of IgM with IgG. This technique uses the RBD of S1 to make SARS-CoV-2 recombinant antigen, and then the fluorescent latex particles modified with this antigen are used as markers. Mouse anti-human IgM and IgG as detection lines, and goat anti-chicken IgY as quality control lines, respectively, and provides rapid results within 10 min after addition of whole blood, serum or plasma samples. In addition, the technique achieved semiquantitative detection with the help of a single channel or multichannel immunofluorescence analyzer, and the sensitivity and specificity measured in 733 COVID-19 confirmed sample compared with RT-PCR were high, 89.22% and 96.86%, respectively. The method was designed in the traditional way of immunofluorescence technique, which also obtained better sensitivity and specificity. However, there are two drawbacks, firstly, it did not use the advantages of the fluorescence technique to achieve the quantification of SARS-CoV-2. Secondly, although many samples were used for validation, they lacked the support of COVID-19-negative samples. Therefore, in combination with the above, the relevant similar products still need to enhance the fluorescent markers and do a comprehensive clinical validation, to achieve a quantitative assay with good performance and simplicity.

Quantum dots (QDs) are widely used to replace conventional fluorescent nanoparticles due to their wide excitation range, good photostability, and high fluorescence quantum yield. When QDs are irradiated by light pulses, they produce a variety of colors to mark the substance to be measured (Chang et al., 2021). In addition, when multiple QDs are irradiated and excited by a laser of a certain wavelength, multiple colors can be observed and multiple measurements can be performed simultaneously (Matea et al., 2017). And it can achieve more accurate quantitative detection compared to the fluorescence detection method. Li et al. (2022) developed a fluorescent LFIA based on QDs nanoparticles to detect the N protein-specific antibodies, representing the majority of conventional QDs for the detection of SARS-CoV-2. This study used ZnCdSe/ZnS QDs-conjugated N proteins as probes, SPA as a capture molecule, and specific antibodies for N protein as target points. If the subject is antibody positive, the complex of specific antibodies bound to the QDs-conjugated N proteins will be captured by the SPA and fluorescence will be shown, and the remaining QDs probe will bind to the antibody at the quality control line, which can be quantified by the fluorescence immunoassay analyzer. The method requires only 1 μl of serum as a sample and can be performed within 20 min. The analysis of five clinical samples showed high sensitivity and good specificity with a detection limit of 48.84 ng/ml. However, the performance advantages over assays such as SERS cannot be demonstrated. However, it was found that some new techniques of QDs for SARS-CoV-2 detection could be borrowed for LFIA, thus providing excellent new options for POCT continuously. For example, a QDs method for the quantitative detection of SARS-CoV-2 IgG reported in the study by Moabelo et al. has an excellent detection limit of 4 pg/ml, which is a cause for concern (Moabelo et al., 2021). The method is a highly sensitive QDs-linked immunoassay based on QDs nanoparticles and magnetic iron oxide (MnFe3O4) nanomicrospheres. With MnFesO4-coupled mouse anti-human IgG as the capture probe and QDs-coupled rabbit anti-human IgG as the detection probe, these three form a sandwich structure thus emitting fluorescence when antibodies are present in the serum (Guo et al., 2020). This method coincides with the principle of LFIA. If the capture probe and detection probe are fixed on the binding pad and detection line respectively, and the substances at the buffer and quality control line are investigated more deeply, it is expected to make more commercial and intuitive LFIA test strips, which also provide another possibility for SARS-CoV-2 related detection.

In addition to this, several methods have been used for the detection of SARS-CoV-2 antibodies. For example, Faezeh et al. (Ghorbanizamani et al., 2021) developed a homemade marker based on the principle of dye-loaded polymersome for anti-body test strips for SARS-CoV-2. In this study, a methoxy polyethylene glycol-b-polycaprolactone diblock copolymers were used to prepare the polymer and loaded with a Komas blue dye, thereby labeling the antibody in the sample. If the result is positive, the detection line, which has been immobilized with the purified antigen, binds to the antibody of the sample due to the principle of competitive LFIA and finally reaches the rabbit anti-human IgG-coated quality control line to bind to the remaining polymer, presenting two blue bands. The assay is diagnostic by visual colorimetry and can be completed within 9 min. By testing 60 clinical samples and comparing them with RT-PCR, it yielded a good sensitivity of 92.2% as well as a false positive rate of 0% and a false negative rate of 15.5%.

Antibody assays have attracted many researchers and are widely used because of their cheap production, low developmental skills, and ease of operation, which has led to a wide variety of methods (Chen et al., 2021b). The detection of different types of immunoglobulins allows us to obtain more information about the duration of infection of the infected person (Mallano et al., 2022). However, antibody testing has several limitations. First, because antibodies are present in the blood, the available data require whole blood, serum, or plasma as the sample for antibody detection, which makes sampling more difficult and makes LFIA much less easy to perform (Harritshøj et al., 2021). Secondly, after antigen invasion, there is a difference in the time of production and duration of different antibodies with a certain lag, so it is not conducive to the initial screening of infected patients (Hakki et al., 2022). In addition, the false-positive rate of antibody testing, especially the false-negative rate, is high compared to that of nucleic acid testing, which is not conducive to accurate diagnosis and precise control of virus transmission. However, researchers have tried to improve the sensitivity and specificity of the test by various methods, and it has been shown that the combined IgM-IgG test is effective in improving sensitivity and specificity due to the increased variety of antibodies detected and the expanded range of antibody production processes (Paces et al., 2020; Shen et al., 2020; Kang et al., 2021b). In addition, as the number of people receiving the SARS-CoV-2 vaccine increases, the need to assess the effectiveness of the vaccine and the body’s resistance to SARS-CoV-2 is high, and antibody testing may be a good method. The choice of different methods for antibody detection has been carried out by scholars with the common aim of finding more sensitive, specific or convenient detection methods.

## 3 LFIA technique for SARS-CoV-2 antigen detection

Antigens are substances that induce the body to produce antibodies, such as most proteins, bacteria, and viruses can be used as antigens. Although, the current LFIA technique of antigen is less studied in the diagnosis of COVID-19, the LFIA technique used for antigen detection, such as Cryptococcus (Zeng et al., 2021), Ebola virus (Wonderly et al., 2019), hepatitis C virus (Reddy et al., 2020), etc., has a certain clinical application basis, so the feasibility of LFIA antigen detection of SARS-CoV-2 exists. Investigations have shown that various proteins such as N, S, and RBD in SARS-CoV-2 as antigenic targets have been applied by some researchers in LFIA techniques.

Colloidal gold has certainly been used in the detection of antigens in LFIA. Peng et al. (2021) modified the traditional method of colloidal gold and developed an LFIA test strip for the rapid detection of NP antigens using colloidal gold nanoparticles after copper deposition. The investigators placed a colloidal gold probe conjugated with mouse anti-N protein monoclonal antibody 1 on a binding pad, and then immobilized mouse monoclonal antibody 2 and goat anti-mouse IgG on the detection and quality control lines, respectively, to form an LFIA test strip. The special feature is that after the reaction of the substance in the test strip is completed, it is immersed in a solution containing Cu2+ and sodium ascorbate, and after a series of reactions copper can be deposited on the strip with colloidal gold binding, thus enhancing the signal. The method can be completed in about 20 min for qualitative detection, while the copper deposition process takes only 3 min, but can amplify the signal to 3 times the traditional one, and its detection limit is 0.01 μg/ml. Thus, it can be seen that LFIA after copper deposition has the advantages of low cost, high efficiency, and high sensitivity, and if it can be further validated and improved in clinical samples, it may become a new choice for SARS-CoV-2 antigen detection. Of course, similar methods are more common, such as silver staining, biotin-affinin system, and enzyme catalysis (Mertens et al., 2020; Ibrahim et al., 2021). All these methods, when combined with the colloidal gold method, can be used to improve the sensitivity of LFIA test strips and can be used as a reference by researchers when developing them.

Immunofluorescence techniques have also been applied in this regard. For example, the commonly used fluorescent microsphere labeling method. Zhang et al. (2020) developed a LFIA test strip based on fluorescent microspheres for the detection of the N protein of SARS-CoV-2. In this study, a fluorescent microsphere monoclonal antibody complex for N protein was prepared and mouse monoclonal antibody and goat anti-rabbit IgG were immobilized on NC membrane as detection and quality control lines, respectively. A nasal or oropharyngeal swab was taken from the subject, and if the result was positive, the N protein in the sample first bound to the fluorescent microsphere monoclonal antibody complex immobilized on the releasing pad, and then to the monoclonal antibody on the detection line to form a double antibody sandwich complex. The remaining fluorescent microsphere-modified complex continues to move forward to the quality control line to bind to the goat anti-rabbit secondary antibody, thus generating a fluorescent signal in both lines, which is finally quantified by a fluorometric LFIA analyzer. In this study, by testing 990 different clinical samples, the specificity of the method was 100.00% and 97.29% for healthy controls and patients with other respiratory diseases, respectively, and the sensitivity was 67.15% and 7.02% for progressive and cured cases, respectively, and the detection limit of the N protein was 100 ng/ml. Some researchers have improved the fluorescent microspheres, thus greatly improving the sensitivity. Mao et al. (2022) developed a SARS-CoV-2 N protein LFIA test strip based on p-toluenesulfonyl-modified fluorescent microspheres. The p-toluenesulfonyl-modified nanomaterials can provide sulfonyl esters, which can covalently link antibodies or other ligands containing primary amino or sulfhydryl groups, allowing the nanomaterials to be more tightly linked to the antibody on the detection line, thus improving the capture rate of the target analyte and achieving a detection limit of 0.01 ng/ml for its test strips. LFIA qualitative test strips can increase the sensitivity by such a large amount without major differences in the use of the test strips, and also have the advantages of being more affordable, which can be used as one of the methods for the development of new crown antigen detection products. However, a certain dose of p-toluenesulfonyl is toxic and can be absorbed through the skin, and developers need to consider such issues, such as the need to explore the issue of dose and toxicity when mass-producing and marketing the product, to protect it well, and to use it in a ventilated area (Metcalf and Kearns, 1941).

Some researchers have also designed a colorimetric and fluorescent dual-mode detection method. For example, Han et al. (2022) developed a dual-mode LFIA test strip based on colloidal gold and QDs for neo-coronavirus S protein detection. Their markers were silica coated with a mixture of a monolayer of 20 nm colloidal gold and QDs. The method has a detection limit of 1 and 0.033 ng/ml for S protein detection by the colorimetric and fluorescence functions of the companion biosensor, respectively. This similar dual-mode method has the advantage of being selectively dual-functional. The visual colorimetric method can be performed first when the working environment and equipment are not ideal or for rapid screening of suspected SARS-CoV-2 infection, etc.; when sensitive and quantitative detection of viral infection is required at the early stage of detection, the accompanying biosensor can be used for fluorescence determination.

Compared with the above methods, SERS still presents a greater advantage. Liu et al. (2022) designed an LFIA test strip based on SERS tags modified with Fe3O4/DTNB@Au/DTNB nanoparticles for the quantitative detection of SARS-CoV-2 antigen. Its probe utilizes magnetic Fe3O4 to adsorb pathogens directly onto the surface. The process is to gradually attach Au nanoparticles to the magnetic Fe3O4 and then modify it twice with DTNB, in which it is immobilized with PVP and NH2OH-HCl. The team developed the design of LFIA test strips for triple testing, taking into account the impact of multiple viruses on disease determination, in which the detection limit of new coronavirus is 8 pg/ml. The obvious sensitivity advantage of the SERS method can be seen from its detection limit, but what attracts us more here is the design of the pathogen probe for triple testing. The team used a magnetic probe to simplify the probe structure and avoid the interference of impurities in the sample. A double DTNB modification was also performed, which, from experimental results and related investigations, correlates with a greatly enhanced detection signal.

In addition to these popular studies above, other methods for LFIA detection of SARS-CoV-2 have also been investigated. For example, An antigen detection LFIA test strip was designed by Faezeh et al. (Ghorbanizamani et al., 2021). This antigen detection method labels the antigen with a polymer prepared from glycol-b-polycaprolactone diblock copolymers loaded with Thomas Brilliant Blue, and the quality control line is immobilized with rabbit anti-human IgG. But differs in that it utilizes the double antibody sandwich principle to capture the antigen by immobilizing a purified antibody at the detection line. In addition, the test requires a nasal swab as the sample, and it takes only about 5 min to complete the diagnosis by the naked eye, and more detailed antigen information can be obtained after instrumental analysis such as electrophoresis and mass spectrometry. By testing 60 clinical samples, the measured sensitivity was 93%, and the false-positive and false-negative rates were 0% and 14%, respectively. Lee et al. (2021) developed a LFIA test strip for the detection of S1 protein. First, unlike other products used for antibody detection, this test strip consists of angiotensin converting enzyme 2 to capture S1 protein, which then binds to S1 antibody to form protein complexes. The study sample was taken from a nasal swab and the detection limit was 1. 86 × 10^5^ copies/mL.

Although there are various probes for antigen detection, most of them use the double antibody sandwich principle. It has many advantages: first, it usually requires only qualitative judgment with the naked eye, and the samples are mainly nasal or oropharyngeal swabs, so the requirements for equipment and sampling are lower and the operation is easier (Safiabadi Tali et al., 2021a). Second, the antigen detection of SARS-COV-2 itself, without a window period, can be used for early screening of infection. However, studies have shown that its sensitivity and specificity are lower than those of nucleic acid and antibody tests, and it increases the probability of false positives and false negatives (Kowada, 2021). In addition, the risk of infection in antigen detection is a problem that cannot be ignored, but it is ideal for point-of-care testing (POCT) because it can be performed with only nasopharyngeal swabs and LFIA kits, and if it is accompanied by relevant monitoring software, it can greatly save labor and material resources, control the spread of the epidemic, and obtain large data related to the epidemic, and it can facilitate self-testing, thus reducing psychological anxiety and worry.

## 4 LIFA technique for SARS-CoV-2 nucleic acid detection

Nucleic acids are carriers of biological genetic information and are divided into ribonucleic acid (RNA) and deoxyribonucleic acid (DNA), which is one of the important indicators for determining SARS-CoV-2 infection. Common LFIA nucleic acid techniques detect nucleic acids through signals generated by combining nucleic acids with capture and detection probes. This method has been used in many clinical applications, such as for the detection of pathogenic microorganisms like *Pseudomonas aeruginosa* (Choi et al., 2016) and hepatitis B virus (Yu et al., 2020), so it can also be tried for the nucleic acid detection of SARS-CoV-2. In addition, with the prevalence of RT-PCR as the gold standard for the diagnosis of COVID-19, many scholars have been thinking of methods that can replace it for more rapid and accurate detection, which has incubated the idea of combining various rapid nucleic acid amplification techniques with LFIA. In addition, recently, the CRISPR-based SARS-CoV-2 nucleic acid detection has received much attention, which combines gene editing technology with traditional LFIA and provides a new idea for nucleic acid detection.

### 4.1 SARS-CoV-2 nucleic acid detection method based on non-CRISPR technology

Zhang et al. (2021) investigated a laminar test paper incorporating reverse transcript loop-mediated isothermal amplification (RT-LAMP) for the visual detection of the *N* and *ORF1ab* genes of SARS-CoV-2. The method divides the detection process into two steps. In step 1, RT-LAMP is applied to efficiently and rapidly amplify RNA without specialized equipment. After treating the sample to be tested with lysate, RT-LAMP-based amplification of fluorescein FITC-labeled dUTP, outer primers biotin-labeled inner primers are performed to generate labeled amplification products. In step 2, LFIA test strips are applied for detection. The paper is inserted into the reaction tube and if the result is positive, the amplification product and the streptavidin-coated particle complex can be captured by the anti-FITC antibody at the detection line and show a red band. The whole detection process can be completed within 40 min, and the amplified test strips only need 3 min to complete the chromatography and obtain the results, and the sample required for the test is a nasal or pharyngeal swab. The detection limits of the *N* and *ORF1ab* genes of SARS-CoV-2 pseudovirus were less than 2 copies/μL, and the results of 12 clinical RNA samples (8 RT-PCR-positive and 4 RT-PCR-negative samples) were compared with RT-PCR and showed 100% concordance. Zhu et al. (2020) devised a combined multiplex reverse transcription loop-mediated isothermal amplification (mRT-LAMP) and lateral flow biosensor (LFB) assay named mRT-LAMP-LFB for the genes of N and ORF1ab to diagnose COVID-19. The approximate reaction procedure is to design two primer sets, with primers for the ORF1ab gene fluorescein-biotin labeling and digoxigenin-biotin labeling for the primers of the N gene; then many double-stranded amplicons with these markers attached can be generated under mRT-LAMP at a constant temperature of 63°C; finally, the resulting solution is then transferred to LFB, the essence of which is LFIA test strips, and the biotin on the amplification products can be bound to dynein streptavidin-coated nanoparticles (SA-DNPs), which can amplify the signal. The anti-fluorescein antibody and anti-digoxin antibody immobilized on detection line 1 and detection line 2, respectively, can capture the amplification product, so that it can be judged under the naked eye, and it is positive when both detection lines and quality control lines show red color. The method is qualitative in less than 1 h and is highly sensitive and specific with a predicted cost of $5.5. It has a detection limit of 12 copies/reaction (24 copies/μL) and does not cross-react with non-SARS-CoV-2 templates. Its accuracy was 100% by testing 33 positive patients and 96 negative patients. What this method shows us, in addition to its excellent performance, is the reliability of the assay after the detection of clinical samples, the accuracy of the results brought by the genetic double detection of N and ORF1ab, the low requirements for operation and instrumentation, and the careful estimation of the cost of the product. And comparing the two LAMP-related methods mentioned above, the advantages do not seem to be comparable. However, some aspects can be considered for improvement, for example: although both perform dual gene assays, the Zhang et al. design does not seem to fully highlight the advantages of the dual gene assay, and one can try to apply different markers to label the two genes as in the method designed by Zhu et al. and also design separate bands for both genes on the test strips, thus making the presented results clearer.

Yu et al. (2020) developed a novel LFIA technique that allows RT-PCR to obtain the desired nucleic acid products for simultaneous detection of *RdRp*, *ORF3a*, and *N* genes. After reverse transcription and amplification, the resulting nucleic acid is added dropwise to the sample pads of LFIA strips and passed successively through the detection line containing capture probes for *RdRp*, *ORF3a*, and *N* genes, and the signal is finally read by a microfluorescence detector. The method was applied to 162 clinical samples and compared with RT-PCR, showing 99.4% concordance and a detection limit of 10 copies/reaction (2 copies/μL).

Chen et al. (2021a) designed a LFIA test strip based on a Duplex Reverse Transcription-Multienzyme Isothermal Rapid Amplification (RT-MIRA) for the visualization of the *N* and *ORF1ab* genes of SARS-CoV-2. The method eliminates the need for RNA extraction and requires only 2 steps for the detection of nasal or pharyngeal swab samples. Firstly, RT-MIRA is applied to amplify the dual target of SARS-CoV-2 in a single reaction. Secondly, the amplification products are captured on LFIA strips by colloidal gold probes made of *ORF1ab* and *N* genes combined with a digoxigenin ligand and a FAM at the 5′ end, respectively, and as the amplification products flow along the strips, the ORF1ab and N genes are successively detected. The *ORF1ab* gene and the *N* gene were detected on the two detection lines immobilized with anti-digoxigenin and anti-FAM antibodies and showed a red band. The method takes only about 25 min to complete the assay, including 3 min of strip chromatography time. The 95% detection limits for the *N* gene and *ORF1ab* gene were 0.049 copies/μl and 0.050 copies/μl, respectively. 243 clinical samples were tested using the RT-MIRA chromatography system, and the results showed 100% agreement with the Real Time Quantitative PCR results.

Zou et al. (2021) combined an isothermal non-enzymatic signal amplification system and LFIA technology to develop a catalytic hairpin assembly-LFIA assay system witch for the detection of the N and ORF1ab genes of SARS-CoV-2.The investigators labeled the 5′ ends of complementary single-stranded hairpin DNA with digoxin and biotin, respectively, to make probes, and sprayed streptavidin and fluorophore Alexa Fluor 647 double-labeled polyethylene nanoparticles on the detection line, thus serving as part of the catalytic hairpin assembly-LFIA detection system. The method uses catalytic hairpin assembly to amplify SARS-CoV-2 RNA extracted from nasal or pharyngeal swabs, and after LFIA test strips, the fluorescence signal is detected by a fluorescence detection device, which ultimately allows quantitative detection within 90 min. The method was applied to 15 clinical samples and compared with RT-PCR, showing 100% concordance and a detection limit of 2 copies/μl.

Recently, antisense oligonucleotides (ASOs) can be used to target the N gene of SARS-CoV-2 (Borghei et al., 2022). Moitra et al. (2020) reported a gold nanoparticle-based turbidimetric assay, in which sulfhydryl-modified ASOs were covered on gold nanoparticles, and when the N gene of SARS-CoV-2 was encountered, the gold nanoparticles selectively aggregated due to their plasmon resonance properties (Jain et al., 2006). Then RNaseH is added to separate the RNA strand from the RNA- Dna heterodimer, which enables the aggregation between the gold nanoparticles again, thus forming the aggregation phenomenon visible to the naked eye, which is judged to be positive for COVID-19. The reaction does not require any complex instrumentation and can yield rapid qualitative results within 10 min, and the experimental limit of detection for RNA with SARSCoV-2 viral load is .18 ng/uL. The advantages of this method in terms of time, portability, and cost effectiveness are undoubtedly outstanding in the genetic testing of COVID-19 and deserve our consideration. In addition this method may provide a reference in the production of similar LFIA test strips, if the modified nanogold particles are attached to the detection line, whether it can also visually show the results visible to the naked eye, and, if the probe can be designed on the binding pad, whether it will increase its specificity, etc. Of course, this is just an idea and further research is needed, but it is certain that if a design similar to LFIA test strips is used, it can promote commercialization and not be discarded in the form of solution after the test is completed, which can improve biosafety assurance. When it comes to ASOs, it is important to mention the electrochemical biosensor chip pioneered by Alafeef et al. (2020) that can perform the assay in an ultra-fast and convenient manner. This is an electrochemical biosensor chip consisting of properly designed ASOs combined with graphene and gold nanoparticles for the detection of the N-gene of SARS-CoV-2, which can be read by hand only in 5 min to produce results with high specificity, sensitivity, and accuracy. The method uses gold nanoparticles covered with optimized concentrations of four ASOs probes to selectively target the viral N gene with a detection limit of 6.9 copies/μl. Moreover, the team found that the sensitivity of ASOs directly affixed to the surface of gold nanoparticles is much less than when covered on the surface, providing a reference value for researchers when designing ASOs-related nano markers. In addition, since the WHO considers the RdRp gene to be relatively more analytical than the N gene in detecting neocrown pneumonia infection (Corman et al., 2020). Kumar et al. (2022) conducted a study on the visual detection of the RdRp gene based on gold nanoparticles. The principle is that hybridization of the oligonucleotide probe of the RdRp gene with the RNA target of SARS-CoV-2 of patients leads to salt-induced aggregation and a pink-to-blue color change of gold nanoparticles for visualization purposes. The method can be completed qualitatively in less than 30 min, and by testing 136 clinically positive samples and comparing them with RT-PCR, it yielded a sensitivity and specificity of 85.29% and 94.12%, respectively, with a limit of detection of .5 ng. This series of oligonucleotides on the probe allows for a significant reduction in detection time, and results can be obtained by simple methods such as colorimetry, with a very low instrument. The personnel requirements are very low, which provides a good reference value for nanoprobes for our related gene assays.

### 4.2 SARS-CoV-2 nucleic acid detection method based on CRISPR technology

**FIGURE 2:**
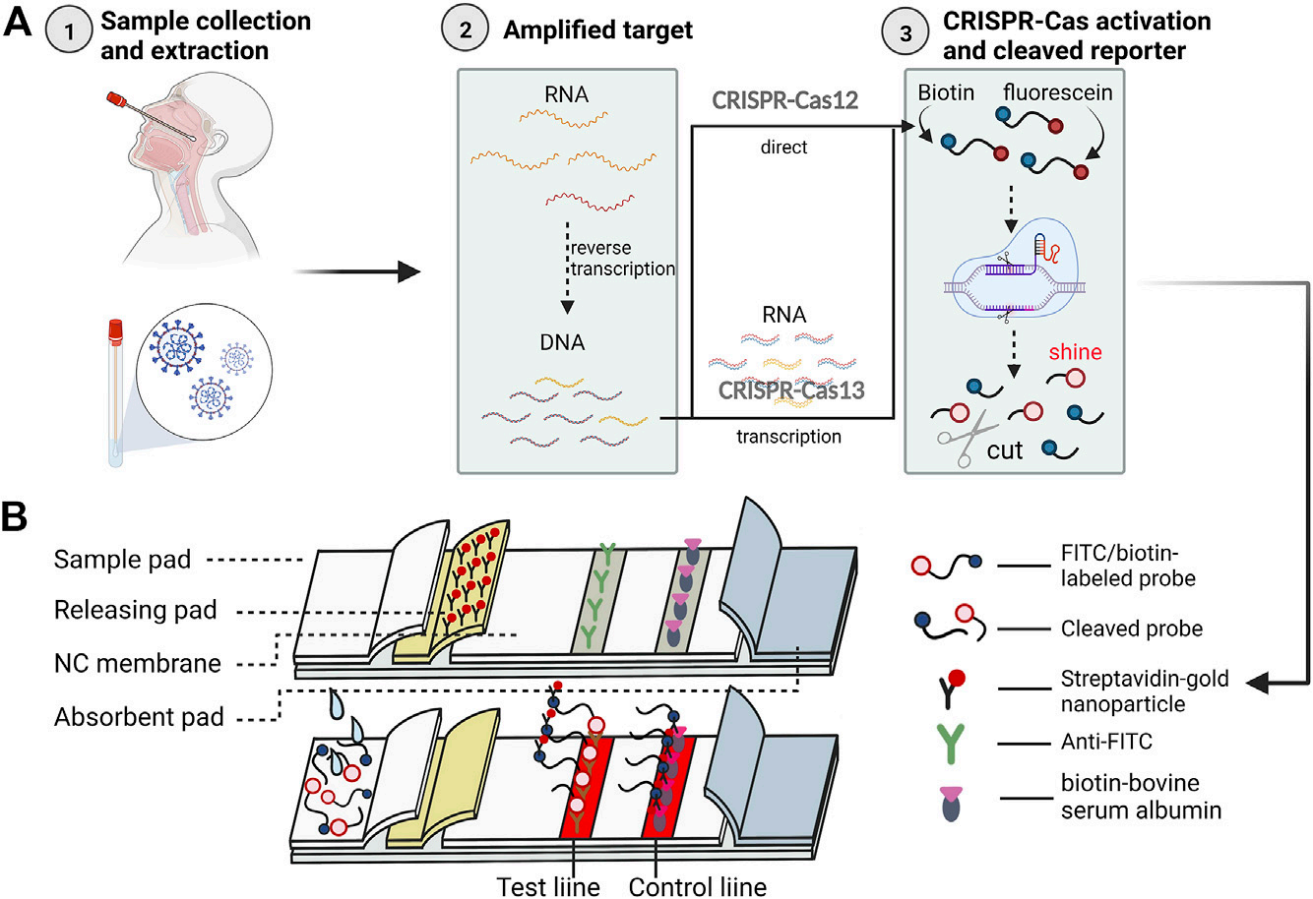
The CRISPR-Cas system for the whole process of COVID-19 immunochromatography detection. Illustrative scheme:The figure briefly summarizes the technical flow of most current CRISPR combined nucleic acid amplification and LFIA using the method designed by Zhu et al. (2021) **(A)** In total, there are three major steps before using immunochromatographic test strips. First, a nasal or pharyngeal swab is used to collect a sample from the subject and extract RNA. second, the nucleic acid is amplified, and the reverse transcription amplification method is generally chosen, in which the extracted RNA is specifically amplified after binding to a probe modified with a fluorescent moiety (fluorescein) and a quenching moiety (biotin), during which the target RNA is converted into a target for more DNA. Then the CRISPR-Cas system is applied to cut the target gene motif, but because Cas12 and Cas13 cut RNA and DNA respectively, the nucleic acid also needs to be transcribed to RNA when Cas13 is selected. Third, the CRISPR-Cas system cuts and a probe with the target RNA or DNA bound to the Cas protein in advance is used to identify the target nucleic acid The CRISPR-Cas system is activated when the target nucleic acid, which has been modified by fluorescent and quenching motifs, binds to the probe and cleaves the nucleic acid sequence non-selectively. The final target nucleic acid sequence is cleaved and the fluorophore fluoresces, allowing the detection of the signal (Ramachandran and Santiago, 2021). **(B)** The test strip located at the top is a schematic diagram of the structure of the product, and the one at the bottom is presented after receiving a positive sample. The above processed products were transferred to LFIA strips for examination, and the cleaved and uncleaved nucleic acid fragments were first labeled with colloidal gold and then flowed to detection and quality control lines immobilized with anti-biotin and anti-luciferin antibodies, respectively, so that the aggregation of uncleaved nucleic acid fragments could show a red band, while the cleaved nucleic acid fragments could be detected by a fluorescence detector. (This figure created with BioRender.com).

CRISPR-Cas is a species-adaptive immune system (Hille et al., 2018) with natural and extraordinary gene editing (Alkhnbashi et al., 2020) and cleavage capabilities for disease (Jolany Vangah et al., 2020) and microbial detection (Medina-Aparicio et al., 2018). Currently, this system has been found valuable by researchers for SARS-CoV-2 nucleic acid detection, which mainly contains studies related to CRISPR-Cas9, Cas12a, and Cas13a (Figure 2). All three systems consist of large single multi-domain proteins (Koonin et al., 2017), and the CRISPR-Cas9 system is widely used to cleave target DNA (Hryhorowicz et al., 2017) *in vitro* by crRNA guidance. In recent years, the discovery of the ability of Cas12a and Cas13a to non-specifically degrade DNA (Zetsche et al., 2015) and RNA (Gootenberg et al., 2017), respectively, upon activation has revolutionized the nucleic acid field (Koonin et al., 2017).

Qin et al. (Zhang et al., 2022) chose a CRISPR/cas13-based fluorescent nanoparticle SARS-CoV-2 detection method and performed compliance experiments. This study combined a cas13-based nucleic acid detection strip with quantum dot microspheres for the detection of the *S* gene of SARS-CoV-2. First, sufficient target nucleic acid was obtained by reverse-transcription recombinase-aided amplification, and then the CRISPR/Cas13 reaction was applied to specifically identify and cleave the amplification product, followed by flowing the cleaved fragment to the detection line, mixing it with sheep anti-fitc IgG-labeled quantum dot microsphere antibody and generating a fluorescent signal, which could finally be detected in a intelligent miniature fluorescence detector. The method requires samples of pharyngeal swabs, anal swabs, or sputum, and the reaction time in the test strips is 15 min, and the overall detection time does not exceed 1 h. After testing 87 clinical cases and comparing with RT-PCR, *p* < .05 was obtained, so the method is not statistically different from PCR, and the sensitivity and specificity are 100%.

Sun et al. (2021) combined reverse transcription and Reverse Transcription and Recombinase Polymerase Isothermal Amplification (RT-RPA) with CRISPR-Cas12a technology to develop a LFIA strip to detect the *N* and *RdRp* genes of SARS-CoV-2. This technique firstly uses RT-RPA to thermostatically amplify the extracted nucleic acids to a certain amount and then uses CRISPR-Cas12a to cleave the FAM-biotin reporter molecule and continue to amplify it, then uses the designed OR-DETECTR to generate a fluorescent signal and finally performs fluorescence detection. The method selected pharyngeal swabs as samples, and OR-DETECTR took about 50 min. After comparing 50 negative samples with PCR, the results were completely consistent, and the detection limit was 2.5 copies/μL. Yu et al. (Wang et al., 2021b) reported the CALIBURN nucleic acid detection platform, which also utilizes RT-RPA with CRISPR-Cas12a technology for the detection of the *E* and *N* genes of SARS-CoV-2, with the entire platform reflecting a process of approximately 1 h. After testing 63 clinical samples using this method and comparing it to RT-PCR, a false-negative rate of 7.9% was obtained; in addition, 57 negative results of 57 negative samples indicated a specificity of 100%.

Zhu et al. (2021) designed a method for MCCD detection of the SARS-CoV-2 N gene and ORF1ab gene based on the RT-MCDA and CRISPR-Cas12a system. After adding primers modified with PAM sites, the method amplifies nucleic acids by RT-MCDA, followed by excitation of the CRISPR-Cas12a/CrRNA system to cleave biotin and fluorescein isothiocyanate (FITC)-modified nucleic acids, and then use the lateral flow biosensor detection. Streptavidin-immobilized gold nanoparticles are immobilized on the releasing pad of the LFIA strips, and anti-FITC and biotin-bovine serum albumin are immobilized on the detection and quality control lines of the NC membrane, respectively, to serve as capture reagents (Figure 2B). The method was able to produce results within 1 h. After testing 114 samples (37 RT-PCR-positive and 41 RT-PCR-negative samples), 100% sensitivity and specificity were obtained when compared with RT-PCR results.

Xiong et al. (2021) combined the CRISPR/Cas9 system with RT-RPA technology to develop a simultaneous rapid triple-linelateral flow assay for the *E* and *ORF1ab* double genes of SARS-CoV-2. In this study, the viral *E* and *ORF1ab* genes were first amplified using the multiplex RT-RPA technique, and the two forward amplification products were modified using biotin and digoxin, respectively; then two sgRNAs (sgRNA1 and sgRNA2) containing variable recognition sequences and constant scaffold sequences were designed for targeting the completed *E* and *ORF1ab* genes, respectively. The 2 Cas9/sgRNAs hybridized to the biotin-labeled *E* gene amplicon and the digoxigenin-labeled *ORF1ab* gene amplicon, respectively, and the hybridization complex flowed through the LFIA test paper with the gold nanoparticles-DNA probe on the releasing pad first hybridized to the gold nanoparticles-DNA probe *via* nucleic acid hybridization. The gold nanoparticles-DNA probe first binds to the scaffold sequence of Cas9/sgRNA by nucleic acid hybridization on the releasing pad and then is captured successively on two detection lines immobilized with streptavidin and anti-digoxin antibodies, from which a red band appears due to the aggregation of gold nanoparticles. The detection limits of the test strips for both *E* and *ORF1ab* genes of SARS-CoV-2 were 4 copies/μl. By testing 64 clinical samples (35 RT-PCR-positive and 29 RT-PCR-negative samples) and comparing them with the RT-PCR results, the negative and positive prediction rates were 100% and 97. 14%, respectively.

LFIA for nucleic acid detection combines the advantages of nucleic acid amplification and LFIA, finding a balance between accuracy and simplicity. It is simpler, shorter, and less costly than RT-PCR, requires a simple sample of the nasal or pharyngeal swabs, and has a high degree of consistency between results. Moreover, like the antigen assay, this method also targets SARS-CoV-2 itself without a window period, making it suitable for early screening of infection. However, compared to antibody and antigen assays, this method still has many shortcomings, such as a long detection time, requires relevant equipment, and cannot be self-tested at home; the development is difficult; it is easy to contaminate and difficult to remove, and produce false-positive results.

At present, CRISPR technology in the detection of LFIA of SARS-CoV-2 mainly combines various simple nucleic acid amplification techniques, and then uses the CRISP-Cas12/Cas13 system to shear the amplification products, and finally uses LFIA to visualize the results, which can be seen that the combination of simple nucleic acid amplification method is widely recognized by scholars, and can take into account the portability and high performance. High performance. In particular, the combination of isothermal amplification technology and CRISPR technology allows amplification to be performed in a thermostatic thermos, greatly reducing the requirement for equipment. In addition, there are also researchers developing amplification-free nucleic acid detection based on the CRISPR-Cas system, which can shorten the detection time and reduce the difficulty of detection (Fozouni et al., 2021). Due to the gene editing function of CRISPR, its related products can have the following advantages: firstly, it can form very small gene fragments, which can make it more sensitive and specific; secondly, if combined with markers and other applications, it can achieve site-specific detection of mutations, while most of the functions of RT-PCR can be achieved and it is more convenient and faster than it (Gootenberg et al., 2017). With the continuous spread of SARS-CoV-2, virus virulence will decrease, mutation types will increase, and infectiousness will increase, so rapid diagnosis must be the best way to control the spread of the epidemic in the future, and only by detecting viral load and mutation types can we effectively grasp the viral transmission and related treatment plan of the diseased (Araf et al., 2022). CRISPR technology has the most comprehensive advantages and the ability to detect viral mutations, so it is necessary to strengthen the research related to CRISPR technology. However, it also has certain R&D difficulties, such as overcoming the problems of isothermal amplification and CRISPR/Cas reactions interfering with each other and being prone to aerosol generation during transfer, which need to be continuously solved by researchers (Sun et al., 2021).

## 5 Comparison of the detection performance of the LFIA strips

This review discusses the specificity (SP), sensitivity (SN), positive predictive rate (PPV), negative predictive rate (NPV), detection limit, and convenience of the test by discussing the performance of some of the widely used LFIA test strips with U.S. Food and Drug Administration emergency use authority, and comparing the advantages and disadvantages for the selection of COVID-19 in different scenarios. It also reflects the overall situation of the performance of the LFIA test strips in the market. Due to the differences in the performance of the test strips by various research teams, the results of this paper are divided into two parts: antibody, antigen, and nucleic acid (Table 1 and Table 2).

**TABLE 1:** Comparison of the performance of some of the LFIC test strips authorized for antibody and antigen detection by the U.S. Food and Drug Administration emergency use.

Classification	Product name	Technology	Target	Characteristic	SP, % (95% CI)	SN, % (95% CI)	PPV, % (95% CI)	NPV,% (95% CI)	Testing time	Equipment needs	Reference
Antibody (2022d)	QIAreach Anti-SARS-CoV-2 Total Test	LFIA	Total S Ab	Qualitative	100 (95.2–100)	100 (88.4–100)	100 (49.2–100)	100 (99.4–100)	10 min	QIAreach eHub	Stieber et al. (2020)
	Elecsys Anti-SARS-CoV-2 S	ELISA	Total S-RBD Ab	Qualitative	99.80 (99.7–99.9)	99.5 (97.0–100)	86.4 (73.3–93.6)	100 (34.2–100)	—	cobas e system analyzers	Muench et al. (2020)
	VITROS Immunodiagnostic Products Anti-SARS-CoV-2 Total Reagent Pack	CLIA	Total N Ab	Quantitative	100 (/)	100 (/)	90.9 (/)	100 (/)	—	VITROS 5600/XT 7600 Integrated Systems	Walker et al. (2021a)
	FREND COVID-19 total Ab	Immumofluorescence + LFIA	Total N Ab	Qualitative	87.5 (66.5–96.7)	88.9 (79.5–94.5)	96.0 (88.0–99.0)	70.0 (50.4–84.6)	After 5 min	FREND System	De Munck et al. (2022)
Antigen (2022b)	INDICAID COVID-19 Rapid Antigen Test	LFIA	N Ag	Qualitative	—	—	85.3 (75.6–91.6)	94.9 (91.6–96.9)	20 min	None	Chiu et al. (2021)
	GenBody COVID-19 Ag	chromatographic digital immunoassay + LFIA	N Ag	Qualitative	100 (96.4–100)	94.0 (87.4–97.8)	93.1 (77.2–99.2)	98.0 (93.0–99.8)	20 min	None	Kim et al. (2021)
	BinaxNOW COVID-19 Ag 2 Card	LFIA	—	Qualitative	99.9 (99.7–100)	52.5 (46.7–58.3)	97.5 (93.8–99.3)	95.6 (94.9–99.3)	15 min	None	Prince-Guerra et al. (2020)
	LumiraDx SARS-CoV-2 Ag Test	Immumofluorescence + LFIA	N Ag	Qualitative	92.1 (89.7–94.1)	90.3 (86.3–93.4)	84.9 (81.0–88.0)	95.1 (93.2–96.5)	—	Related fluorescent latex signal detectors	Bianco et al. (2021)

**TABLE 2:** Comparison of the performance of some of the LFIC test strips authorized for nucleic acid (2022c) by the U.S. Food and Drug Administration emergency use.

Product name	Technology	Target	Characteristic	PPV,% (95% CI)	NPV,% (95% CI)	Detection limit, copies/μL	Testing time	Reference
ID NOW COVID-19	Isothermal nicking enzyme amplification reaction	*RdRp* gene	Qualitative	94.5 (85.1–98.1)	99.3 (96.4–99.9)	.511	5–13 min	Babic et al. (2021)
Sherlock CRISPR SARS-CoV-2 kit	RT-LAMP,CRISPR-Cas13a system	*N* and *ORF1ab* gene	Quantitative	100% accuracy compared to RT-PCR		6.75	50 min	Khan et al. (2021)
SARS-CoV-2 RNA DETECTR Assay	RT-LAMP,CRISPR-Cas12 system	*N* and *E* gene	Qualitative	95 (/)	100 (/)	10	45 min	Broughton et al. (2020)
Cue COVID-19 test	Isothermal nucleic acid amplification tests technology	*N* gene	Qualitative	91.7 (/)	98.4 (/)	—	After 20 min	Donato et al. (2021)

The data in both tables below are compared to the gold standard RT-PCR results. Although there is a small difference in cycle threshold values of the samples measured by each experiment, the comparison of the data has high value. Table 1 shows the results that analyzed a certain number of adults or children. The convenience of the assay is reflected by the time required to add the sample and the time required to read the results; the equipment requirements refer to the need for ancillary testing equipment in addition to the LFIA strips. Table 2 shows the results that analyzed a certain number of symptomatic or asymptomatic patients with COVID-19 or other respiratory infectious diseases. The time reflects the convenience of the assay, specifically the time between the addition of the sample and the reading of the results, but the nucleic acid test products all require supporting equipment and are technically demanding. In addition, it presents information about the product in terms of the R&D company, the test target, and the amplification or gene editing technology, which gives some insight into the product being compared and may also help people to keep track of the market.

WHO indicates that it recommends COVID-19 assays with a sensitivity of ≥ 80% and specificity of ≥ 97% (Peeling et al., 2021). Therefore, our team used this as a measure and selected data from the relevant studies with the largest known sample sizes for analysis for reference. First, although the above products utilize different markers, many are not commercially available to describe their specific composition and rationale and therefore are of little reference significance in the discussion. Secondly, we have performed the following analyses by examining the differences in detection principles. Most of the antibody and antigen assays are LFIA-related applications, which can be seen to be very popular in the market, and the principles of such assays are qualitative products. It can also be found that it is often used in combination with other methods such as immunofluorescence, and the methods used in combination have associated supporting detection equipment, although more complex than the visual colorimetric method and more demanding equipment, its performance, especially the sensitivity has been significantly improved. This is probably because the combined method draws on the common advantages of both, optimizes the markers, and is supported by the equipment, and its detected signal is certainly larger than that of visual colorimetric methods. The remaining methods are ELISA and chemiluminescence, of which ELISA does not show significant advantages, while chemiluminescence is the best of the products used and is attributed to its markers, reaction system, and supporting equipment, which gives it the ability to be quantified, thus effectively improving the relevant performance. Moreover, the amount of samples collected is not too small and the variety is comprehensive, so it can be used as a more reliable method for POCT (Walker et al., 2021b). Among the nucleic acid detection methods, a variety of principles are used, and their overall performance is basically better than that of antigen and antibody detection methods and following WHO recommendations, however, the isothermal nicking enzyme amplification reaction method is qualitative, but its detection limit and detection time have great advantages. In addition, the choice of detection target is also closely related to the performance of the product. Among them, antibodies and antigens for N protein and N gene are the most selected, which is also associated with its high stability, where the advantage of multiplex detection is shown in nucleic acid detection, and the performance of multi-gene detection is better than that of a single gene, in addition, it is also mentioned above that RdRp gene is more capable of analyzing new coronavirus infection, so this is most likely the reason for its low detection limit, so the choice of a target can be considered depending on the situation The choice of RdRp gene. Overall, qualitative products currently dominate the market, and their purpose is generally for rapid home detection of COVID-19, without more comprehensive quantitative or mutational analysis, so products at this stage are more inclined to improve convenience while ensuring performance.

## 6 Discussion

Above all, LFIA test strips for SARS-CoV-2 antibody, antigen and nucleic acid detection are designed in a variety of ways. Researchers usually spend their efforts on the selection of many assays and markers, depending on their detection principles, mainly to achieve the ultimate goal of improving the performance and social value of the assay.

First, antibody-related assays often choose colloidal gold in the choice of markers, in addition to fluorescent microspheres, QDs, and dyes; the assays include immunofluorescence, QDs, chemiluminescence, and SERS, in addition to the commonly used colorimetric methods. By analyzing the assay performance, antibody assays are often qualitative, although QDs, chemiluminescence, and SERS methods are commonly used for quantification. The sensitivity of antibody detection is around 90%–95%, the specificity is around 95%–100%, and the detection limit for quantification varies from 1pg/ml-1 ng/ml, with the SERS method performing the best. Speaking of its application, because antibodies need to be produced gradually after the body has been stimulated by the pathogen for some time, with a window period, and because many people have now been vaccinated and their bodies produce antibodies, the detection of antibodies is not a mainstream method for the diagnosis of COVID-19. However, in the future, we can mainly carry out matters such as the evaluation of the duration of infection and the potency of antibodies in patients. From this point of view, the double test and quantitative test of IgM and IgG are very necessary, and we can continue the research from the aspect of SERS or QDs.

Second, the detection of COVID-19 using antigens is a direct test with no window period. There are various methods, still colorimetric, fluorescence, QDs, and SERS methods. Performance, sensitivity, specificity, etc. are lower than those of antibodies. And the qualitative study of antigen detection started long ago, and the method has become mature, and recently gradually changed to quantitative research, but such studies, although the results are good, lack clinical samples to verify, so the follow-up should continue. Because of the current epidemic situation, China and other places are still trying to control the epidemic, and some places are gradually liberalizing their policies, and there is no telling when the post-epidemic era will return, which makes home self-testing and POCT very important directions for development. Among them, home self-testing generally requires less comprehensive results and there is no instrument to perform quantitative testing, so it is recommended to use relevant qualitative methods; POCT for clinical use often requires more reliable and comprehensive results, so quantitative testing is preferable.

Third, the nucleic acid test also has no window period and can be used for the diagnosis of neo-coronaviruses. Compared with the antigen test for SARS-CoV-2, the most significant change is that the performance of each has been improved, and the requirements for instruments, environment, and personnel have been upgraded, so it is intermediate between the gold standard and the antigen test, which is only applicable but very suitable for community or clinical POCT testing. The main methods of nucleic acid detection are an amplification of target genes, often by RT-LAMP, RT-PCR, RT-RAA, RT-PAR, RT-MIRA, and CHA, followed by cleavage or no cleavage by CRISPR technology, and then the reaction products are moved to LFIA for visualization and qualitative or quantitative detection. Their detection limits are around 2–20 copies/μl, and some can be lower than 0.05 copies/μl. In addition, there are a few non-amplified assays that are commonly used for ASOs or cut directly by CRISPR technology. The markers used in these assays are similar and relatively plain, often colloidal gold or fluorescein, digoxigenin-biotin.

Afterwards, based on the discussion of the different assays, we can again find the markers commonly used in these methods and the advantages of each. Four of the most commonly seen methods in the text will be listed here. The first is the colorimetric method, in which commonly used markers must be colloidal gold and products derived from colloidal gold, such as biotin-labeled colloidal gold and copper deposited after the signal can be enhanced, although which substance is better, but also need to be compared in specific cases. And other methods can be combined with colloidal gold to achieve the visualization of the results of the very convenient operation. In addition, colloidal gold is also commonly used in the SERS method. The second is the immunofluorescence method, the commonly used markers for fluorescent microspheres, some researchers have reported p-toluenesulfonyl modified fluorescent microspheres, so the sensitivity is greatly increased. The third is the QDs method, whose commonly used markers are QDs, although less often modified with QDs, although its combination with magnetic MnFe3O4 can make the detection limit greatly reduced. The fourth is the SERS method, which requires binding to metal ions and therefore uses modified Au or Ag as markers in addition, such as 4-NBT-modified Au, double-layer DTNB-modified SiO2@Ag, and double-layer DTNB-modified Fe3O4@Au, etc. The detection limits of the two markers using the double-layer DTNB modification are similar and extremely low. Then we compared the lowest detection limits of the four methods, which were: colloidal gold after copper deposition—0.01 μg/ml; p-toluenesulfonyl-modified fluorescent microspheres—0.01 ng/ml; QDs-conjugated magnetic MnFe3O4 --4pg/ml; double-layer DTNB-modified SiO2@Ag—1 pg/ml. Thus the related colorimetric and fluorescent immunoassays are more suitable for the antigen detection of SARS-CoV-2 in R&D homes, and the QDs and SERS method is more suitable for quantitative antibody detection of SARS-CoV-2, and high standard of antigen and nucleic acid clinical POCT. In addition, the probes for nucleic acid assays are more rudimentary and their performance is often more related to the design of the primers.

Finally, according to the comparison of the situation of the marketed products for SARS-CoV-2 detection with the LFIA products under study, it can be found that the detection methods, most of the marketed products choose conventional colorimetric, fluorescent, RT-LAMP, and other methods. Secondly, some products with relevant clinical sample validation are compared, in which the new products under study that have been clinically validated use the traditional assay methods. The performance of the marketed products was not as good as that of the new products under study, except for antibody detection products, while antigens and nucleic acids were comparable to the marketed products in the comparison. In addition, the performance of antibody assays in marketed products tends to be lower than that of antigen and nucleic acid assays, especially for negative predictive values, but new products under investigation show the opposite. In response to the above analysis, the most likely reason for the low negative predictive value of the antibody test-related products is that the test was performed after vaccination. The difference in the performance of antibody and antigen assays is most likely because the antibody products under study are better designed in terms of markers, etc., and that the research is still basically theoretical while there are still problems with the actual window period, and the clinical validation of the products is less comprehensive than that of the marketed products. Finally, QDs, SERS, and other products with excellent performance have not been clinically validated, and it is hoped that researchers can screen good technologies from them to utilize them in practice so that the detection technology of new coronaviruses can be more prosperous. In addition, there are listed products and research products that have several common features worth mentioning, the detection of the RdRp gene and the use of related enzymes in nucleic acid amplification technology can greatly improve the detection performance.

## 7 Conclusion and outlook

The LFIA technique is simple, inexpensive, and suitable for the rapid detection of SARS-CoV-2. Currently, several flowmetric immunochromatographic assays have been reported for different targets of SARS-CoV-2 antibodies, antigens, and nucleic acids, and we illustrate the current development and future directions of LFIA technology by listing some of the typical methods. The advantages and disadvantages of the methods and markers used for the three types of tests: antigen, antibody, and nucleic acid, are discussed to provide a framework and reference for researchers in the development of LFIA test strips for SARS-CoV-2. Finally, we summarize the advantages and disadvantages between the three different targets and their application scope, as shown in Table 3.

**TABLE 3:** Differences, advantages, and disadvantages of LFIA for antibody, antigen and nucleic acid detection.

	Antibody detection	Antigen detection	Nucleic acid detection
Target	Antibodies produced by the immune response	Related structural proteins	Related structural proteins
Sampling method	Whole blood, serum, or plasma	Nasopharyngeal swabs, sputum, etc.	Nasopharyngeal swabs, sputum, etc.
Methodology development	Nano-labeling technology, followed by gene recombination and protein purification technology to prepare specific recombinant antigens, while screening anti-human IgM and anti-human IgG and other capture antibodies	Screening of paired high-valence monoclonal antibodies, design of optimized process system using various nano-labeling techniques and double antibody sandwich method for rapid detection of antigens in specimens	RT-PCR, RT-MIRA and RT-LAMP were used to amplify pathogenic nucleic acids, and the amplification products were also detected by labeled probes
Application stage and scope	5–14 days after the organism is infected with SARS-CoV-2; on-site testing at hospitals, isolation sites, etc., POCT	Early stage of infection (0 days after the appearance of symptoms) to the body’s elimination of the virus after recovery; on-site testing at hospitals, isolation sites, etc., POCT, home self-test	Early stage of infection (0 days after the appearance of symptoms) to the elimination of the virus by the organism after recovery; on-site testing at hospitals, isolation sites, etc., POCT
Testing time	10–20 min	5–15 min	The faster 25 min or so, most need 1h, while the slow need 2 h
Performance	High sensitivity, high specificity	Lower sensitivity and higher specificity	High sensitivity and specificity
Facilities, environment, and personnel requirements	Lower price of equipment; general biosafety laboratory, on-site testing; easy to operate, lower technical requirements for testing personnel	Mostly qualitative detection, only need to measure the LFIA kit related products, no special equipment requirements; general biosafety laboratory, on-site testing; easy to operate, low technical requirements for testing personnel	More equipment required, slightly more expensive; requires higher laboratory standards; cumbersome operation, high technical requirements for testing personnel
Testing costs	Relatively low	Low	Higher, but lower than RT-PCR
Clinical significance	Detection and analysis of infection status and stage, epidemiological surveys, etc.	Rapid, early screening of suspected COVID-19 cases and asymptomatic infected patients, complementary diagnosis, mass screening in hospitals and cities, means of detection in poor areas	Rapid diagnosis of COVID-19, confirmation of variant strains

Currently, LFIA techniques for antibodies, antigens, and nucleic acids have their characteristics and advantages, but it is difficult to integrate the four aspects of accuracy, convenience, rapidity, and affordable price. Therefore, in the future COVID-19 assay, we can consider switching different detection modes according to different application scenarios, and this method is very suitable for the actual situation and future development direction, and we hope that it can be carried out systematically. Specifically, the detection of antibodies is suitable for determining the duration of infection and the assessment of antibody potency in patients, and highly sensitive probes or SERS, QDs, and other methods can be selected for quantitative studies; antigen detection should play its greatest advantage of portability and carry out qualitative home detection with high sensitivity and specificity in colloidal gold and other aspects; the advantages of nucleic acid detection are mainly good performance, fast detection time compared with gold standards and low instrument requirements, suitable for the community and hospital-based POCT testing with high requirements. Finally, the related application of CRISPR technology in nucleic acid detection has a good prospect for development because of its good performance and its ability to cut genes and analyze SARS-CoV-2 gene and variant strains with technical support, which is the closest to the gold standard and the most comprehensive detection method at present.

Although, RT-PCR remains the current gold standard for the diagnosis of COVID-19, LFIA test strips also have promising applications. If a high-performance and less operationally and equipment-demanding CRISPR method LFIA system can be developed, it is expected to be the most representative method for clinical POCT, which can provide rapid and reliable results for diagnosis, analysis, and dosing of COVID-19. According to a survey of the literature on the use of CRISPR for SARS-CoV-2 nucleic acid LFIA, both Cas12 and Cas13 systems can be used for this assay, and the combination of the two systems is expected to improve performance by obtaining more comprehensive information on the SARS-CoV-2 gene at the time of detection, although the non-specific shearing of the gene by both systems may disrupt. However, since both systems are non-specific for gene shearing, they may destroy other targets to be tested, and interference between analytes and cross-reactivity pose challenges for multiplex assays. In addition, the use of isothermal amplification has greatly reduced the dependence of nucleic acid detection on instruments, and CRISPR technology continues to try to integrate with isothermal amplification technology. We hope that researchers can make good use of isothermal amplification technology and overcome the problems in the direction of multiplex detection technology of Cas12 and Cas13 systems, to develop the POCT test strips of SARS-CoV-2 based on CRISPR nucleic acid chromatography technology, and truly achieve rapid, sensitive, convenient, and specific detection of SARS-CoV-2, to promote the development of COVID-19 diagnosis and contribute our modest contribution to the world’s fight against epidemics.
